# Expression of RNA polymerase I catalytic core is influenced by RPA12

**DOI:** 10.1371/journal.pone.0285660

**Published:** 2023-05-11

**Authors:** Brittany L. Ford, Ting Wei, Hester Liu, Catherine E. Scull, Saman M. Najmi, Stephanie Pitts, Wenjun Fan, David A. Schneider, Marikki Laiho

**Affiliations:** 1 Drug Research Program, Faculty of Pharmacy, University of Helsinki, Helsinki, Finland; 2 Department of Pharmaceutical Biosciences, Faculty of Pharmacy, University of Helsinki, Helsinki, Finland; 3 Department of Radiation Oncology and Molecular Radiation Sciences, Johns Hopkins University School of Medicine, Baltimore, MD, United States of America; 4 Department of Biochemistry and Molecular Genetics, University of Alabama at Birmingham, Birmingham, AL, United States of America; University of South Florida, UNITED STATES

## Abstract

RNA Polymerase I (Pol I) has recently been recognized as a cancer therapeutic target. The activity of this enzyme is essential for ribosome biogenesis and is universally activated in cancers. The enzymatic activity of this multi-subunit complex resides in its catalytic core composed of RPA194, RPA135, and RPA12, a subunit with functions in RNA cleavage, transcription initiation and elongation. Here we explore whether RPA12 influences the regulation of RPA194 in human cancer cells. We use a specific small-molecule Pol I inhibitor BMH-21 that inhibits transcription initiation, elongation and ultimately activates the degradation of Pol I catalytic subunit RPA194. We show that silencing RPA12 causes alterations in the expression and localization of Pol I subunits RPA194 and RPA135. Furthermore, we find that despite these alterations not only does the Pol I core complex between RPA194 and RPA135 remain intact upon RPA12 knockdown, but the transcription of Pol I and its engagement with chromatin remain unaffected. The BMH-21-mediated degradation of RPA194 was independent of RPA12 suggesting that RPA12 affects the basal expression, but not the drug-inducible turnover of RPA194. These studies add to knowledge defining regulatory factors for the expression of this Pol I catalytic subunit.

## Introduction

RNA Polymerase I (Pol I) is a multi-subunit enzyme that operates in the nucleolus of the cell [1–3]. The key enzymatic activity of Pol I is to transcribe the ribosomal DNA (rDNA) into the 13 kb long 47S precursor ribosomal RNA (rRNA), which is the first step of the complex, resource and energy-consuming process of ribosome biogenesis [1, 2, 4]. Ribosome biogenesis is especially critical at times requiring extensive protein synthesis, such as cellular divisions during development and regeneration or for highly specialized functions within differentiated cells [5–7]. In this process transcription by Pol I is the rate limiting factor [1, 2, 8]. The magnitude of Pol I transcription and related metabolic activity in cells underscores the importance of this enzyme; up to 60% of all transcription in cells is by Pol I [4].

Certain disease states such as cancer require constant production of proteins to maintain the demands for pervasive growth and proliferation by the cancer cell [3, 7, 9]. This dependence on ribosome biogenesis manifests by aberrant activation of Pol I in cancer cells and predisposes them to perturbations in this pathway [3, 9, 10]. The addiction to Pol I has uncovered a vulnerability in cancer that creates a new target for therapeutic development. We have discovered a small-molecule compound, BMH-21, which inhibits Pol I in a unique and specific way leading to potent reduction of cancer cell growth without causing DNA damage [11–14]. BMH-21 inhibits Pol I transcription initiation, promoter escape and elongation [12, 14–16]. As a consequence, the largest catalytic subunit of Pol I, RPA194 (RPA1) is degraded in a proteasome-dependent manner [12, 14, 17]. Here we show evidence that RPA194 is stabilized by the small, Pol I-specific subunit RPA12.

RPA12 is crucial for several steps in Pol I transcription cycle, namely initiation, elongation and termination [18–22]. RPA12 has homology to two Pol II transcription factors, Rbp9 and TFIIS [23, 24]. The C-terminus of RPA12, which is homologous to TFIIS, stimulates the cleavage of the nascent RNA strand; while the N-terminus, homologous to Rbp9, is required for RPA12 association to the enzyme core and supports the RNA cleavage activity [18, 25–27]. This cleavage activity is required at transcription termination to release the precursor rRNA [18]. RNA cleavage also supports enzyme proofreading and enables backtracking when the enzyme faces elongation blocks [20, 27]. Elongation blocks such as those produced by errors in nucleosome clearance, nucleotide mismatch or transcription inhibitor BMH-21 can lead to polymerase pausing and challenge polymerase processivity [21, 28–30]. Remarkably, deletion of *RPA12*.*2* in yeast leads to loss of transcription fidelity [27, 31]. Paused Pol I can backtrack up to 20 nucleotides where RPA12 cleavage of the nascent RNA realigns the RNA 3’ end within the active site [26, 27, 29, 30]. These backtracked polymerases can then reinitiate transcription elongation, resolving the stalled state instead of dissociating or being tagged for degradation. In contrast, unresolved elongation blocks activate proteasome-dependent degradation of the RNA polymerases [32, 33]. Interestingly, this conserved mechanism affects only the catalytic subunits and is shared between Pols I, II and III [3, 12, 34–36].

Since Pol I transcription inhibitor BMH-21 causes elongation stress and also affects RPA194 stability, we sought to determine whether RPA12 affects RPA194 turnover. We assessed the effect of RPA12 on Pol I transcription activity and on the localization, expression, and occupancy of RPA194 on the rDNA gene locus. We find that RPA12 affects the basal expression of RPA194 but has minor impact on the drug-inducible turnover of the protein. Despite the decrease in RPA194 expression in RPA12 knockdown cells, Pol I transcription activity and its occupancy on the rDNA remains unaffected, which suggests robust compensation mechanisms for the decreased expression of this critical subunit.

## Materials and methods

### Cell culture and reagents

A375 melanoma (CRL-1619) cells were purchased from American Type Culture Collection. This cell line was authenticated using short tandem repeat analysis by Johns Hopkins Genetic Resources Core Facility. Mycoplasma testing was conducted periodically using qPCR with negative results. The cells were maintained in a humidified atmosphere comprising of 5% CO2 at 37˚C. A375 cells were cultured in DMEM supplemented with 10% fetal bovine serum and 4.5 g/L glucose. Pol I inhibitor BMH-21 (12H-benzo[g]pyrido[2,1-b] quinazoline-4-carboxamide, N-[2(dimethylamino)ethyl]-12-oxo) used in this study was synthesized as described by Colis et al. and verified for purity using liquid chromatography/ mass spectrometry and 1H nuclear magnetic resonance [13]. Yeast cells were grown at 23°C on Yeast Peptone Dextrose (YEPD) agar plates supplemented with indicated concentrations of BMH-21.

### RNA interference

For RNAi using small interfering siRNAs, cells were transfected with 10 nM of targeting gene or negative control siRNAs using Lipofectamine RNAiMAX (Invitrogen), and the cells were incubated for 48–72 hr. The following siRNAs were used: negative control #1 siRNA, RPA12 (s26941 and s26943), and RPA135 (s38603 and s38605) (Ambion, Thermo Fisher Scientific). The following shRNA constructs against RPA12 were obtained from the Johns Hopkins University High-Throughput core: pLKO-shRNA-ZNRD1-19074, pLKO-shRNA-ZNRD1-19075, pLKO-shRNA-ZNRD1-19076, pLKO-shRNA-ZNRD1-19077, pLKO-shRNA-ZNRD1-19078.

### Immunofluorescence

Cells grown on 13 mm glass coverslips were fixed in 3.5% paraformaldehyde or 3.5% paraformaldehyde and 1% glyoxal (40% w/v in water), then permeabilized with 0.5% NP-40 lysis buffer (50 mM Tris-HCl [pH 7.5], 150 mM NaCl, 0.5% NP-40, and 50 mM NaF), and blocked in 3% BSA [12, 37]. The following primary antibodies were used: RPA135 (mouse monoclonal 4H6 1:100; sc-293272 Santa Cruz Biotechnology), RPA194 (mouse monoclonal C-1 1:100; sc-48385 Santa Cruz Biotechnology), PAF53 (rabbit polyclonal 16145-1-AP 1:200; ProteinTech), CAST (rabbit polyclonal PA5-27632 1:200; Bethyl Laboratories), ZNRD1 (RPA12) (mouse monoclonal D10 1:100; sc-393406 Santa Cruz Biotechnology) and fibrillarin (rabbit polyclonal 1:500; ab5821 Abcam). Secondary antibodies used were Alexa 488 and Alexa 594-conjugated anti-mouse and anti-rabbit antibodies (Invitrogen). DNA was stained with Hoechst 33342 or 33258. Images were captured using DM6000B wide-field fluorescence microscope (Leica) equipped with a Hamamatsu Orca-Flash 4.0 V2 sCMOS camera and LAS X software using 40X/1.25–0.75 HCX PL APO CS oil and 63X/1.40–0.60 HCX PL APO Lbd.bl. oil objectives or Zeiss AxioImager.Z1 epifluorescence microscope with EC Plan-Neofluar 63X/1.25 oil objective, Axiocam 702 camera and Zen Blue 3.4 software. Image analysis was conducted using ImageJ to determine the number of pixels and the intensity of the pixels of each protein. These areas over DNA area were calculated. Means of these ratios per treatment were used in the analyses. Each analysis was conducted on average of more than 50 cells and are indicated in the figure legends.

### Immunoblotting

Cells were lysed in RIPA lysis buffer (50 mM Tris-HCl [pH 7.5], 150 mM NaCl, 1% NP-40, 0.1% SDS, and 1% sodium deoxycholate) supplemented with protease inhibitors (Roche), sonicated, and centrifuged at 13,200 rpm for 15 minutes. Protein concentrations were measured using Dc-Protein Kit (Bio-Rad) or BCA Kit (Thermo Fisher Scientific) and normalized against buffer only. Equal amounts of protein were separated on SDS-PAGE, transferred to PVDF membranes (Millipore Sigma), probed for target proteins, and detected using ECL (Perkin Elmer). The primary antibodies used for detection were RPA194 (C-1 1:500; Santa Cruz Biotechnology), RPA135 (goat polycloncal H-15 1:200; sc-17914 Santa Cruz Biotechnology), ZNRD1 (RPA12) (mouse monoclonal D10 1:200; Santa Cruz Biotechnology), DDK (mouse monoclonal TA50011 1:1000; Origene), α-tubulin (mouse monoclonal 10D8 1:1000; sc-53646 Santa Cruz Biotechnology), and GAPDH (rabbit monoclonal 14C10 1:5000; mAb#2118 Cell Signaling Technology). Horseradish peroxidase (HRP)-conjugated secondary antibodies were from DAKO or Santa Cruz Biotechnology. Chemiluminescence images were acquired using Bio-Rad ChemiDoc. Protein densitometry analysis was conducted using ImageJ software and normalized against loading control.

### Immunoprecipitation

Cells were harvested in RIPA lysis buffer supplemented with protease inhibitors (Roche). Protein lysates (1 mg) were precleared using Dynabeads Protein G beads (Invitrogen 10003D) for 1 hour at 4˚C and centrifuged at 5,000 rpm for 5 minutes at 4°C. Primary antibodies (2 μg) against RPA194 (C-1; Santa Cruz Biotechnology), RPA135 (H-15; Santa Cruz Biotechnology) or control anti-mouse IgG (Millipore Sigma) were incubated with the supernatant overnight with rotation at 4˚C. The protein-antibody mixture was collected on Dynabeads Protein G beads (50 μL per sample) for 45 minutes at 4°C followed by 5 washes with RIPA buffer with protease inhibitor (Roche). The beads were resuspended in 2x Laemmli Sample Buffer with DTT and boiled for 10 minutes. Samples were run on SDS-PAGE gel and transferred to PVDF membranes (Millipore Sigma) followed by immunoblotting.

### RNA isolation and quantitative polymerase chain reaction

qPCR was conducted as described in Pitts et al. [17]. RNA was isolated using Qiagen RNease Mini-Kit and 260/280 and 260/230 ratios were measured using NanoDrop. RNA was reverse transcribed using dNTPs (10 mM each), Random Hexamers (50 μM) (Invitrogen), Oligo DT and SuperScript™ II Reverse Transcriptase (Invitrogen). cDNA was mixed with iTaq Universal SYBR Green Supermix (Bio-Rad), Precision Blue™ Real-Time PCR Dye (Bio-Rad) and primer pairs. Amplification was conducted on a Bio-Rad CFX384 Real-Time System C1000 Touch Thermal Cycler.

Primer pairs used were: 5’ETS (forward GAACGGTGGTGTGTCGTT, reverse GCGTCTCGTCTCGTCTCACT), 18S (forward CCCGAAGCGTTTACTTTGAA, reverse CGGTCCAAGAATTTCACCTC), GAPDH (forward GGCCTCCAAGGAGTAAGACC, reverse AGGGGTCTACATGGCAACTG), RPA12 (forward GGCGGTTGTACATTTGGTCT, reverse AATAAGGGATGGGACCAAGG), RPA135 (forward CACAACCAGAGTCCACGGAACA, reverse TCACCAAGGGACTCTGAGGAGT), RPA194 (forward GCGTGGTGACTCCGGGCTTG, reverse CAGGCCGTTTGCCGATGGGT).

### Chromatin immunoprecipitation

Cells were fixed with 1.1% formaldehyde for 6 minutes, washed with PBS, scraped, normalized according to cell numbers, and pelleted at 4°C using 500 x g. Lysis was completed using the iDeal ChIPseq kit (Diagenode, Cat# C01010170). Following chromatin isolation, chromatin was resuspended in iS1b buffer (Diagenode) and sheared using Covaris ME220 Focused-ultrasonicator. Immunoprecipitation was conducted using POLR1A/RPA194 (C-1; Santa Cruz Biotechnology) (5 μg) antibodies for 6 hours and the precipitates were collected on Dynabeads G beads (Thermo Fisher) at 4°C. The beads were washed, and the chromatin was eluted and purified. qPCR was conducted as above.

Primer pairs used were: Promoter -48 (forward GAGGTATATCTTTCGCTCCGAGTC, reverse CAGCAATAACCCGGCGG), 5’ETS 851 (forward GAACGGTGGTGTGTCGTT, reverse GCGTCTCGTCTCGTCTCACT), 18S 4446 (forward CCCGAAGCGTTTACTTTGAA, reverse CGGTCCAAGAATTTCACCTC), Terminator Tr1 13508 (forward ACCGCGGCCTTCTCCA, reverse TGCGGTTCGTCCCGAC), Terminator Tr2 15364 (forward GCCGTCAGCCAGTAATGCTT, reverse GAAAACGCAAGGCAAAACCA), IGS 30541 (forward ACTGGCGAGTTGATTTCTGG, reverse CGAGACAGTCGAGGGAGAAG).

### Northern blotting

Cells were lysed in TRIzol and RNA was isolated using Purelink RNA mini kit (Themo Fisher). RNA concentration and purity were measured using NanoDrop. Equal amounts of RNA were separated on 0.8% agarose 1% formaldehyde/MOPS [3-(N-morpholino)-propanesulfonic acid] gels. RNA was then transferred to Hybond N+ nylon membranes (Amersham Pharmacia Biotech) and probed with radiolabeled oligonucleotides [38]. The Northern blots were first probed with ITS1 probe, exposed, stripped by boiling in 0.15 M NaCl, 0.0015 sodium citrate, 0.1% SDS and the probed with a 28S rRNA probe. After each probe, Northern blots were dried and visualized using phosphorimage analysis (Typhoon 5, GE Life Science). Probe sequences used were ITS1: 5′ AAGGGGTCTTTAAACCTCCGCGCCGGAACGCGCTAGGTAC 3′, 28S: 5′ CTTTTCCTCCGCTGACTAATATGCTTA 3’.

### Statistical analysis

The following statistical methods were conducted using a minimum of three independent biological replicates: Analysis of variance (ANOVA), Student’s two-tailed t test and non-parametric Mann-Whitney two-tailed t tests, using Excel or GraphPad Prism software. The test used for each experiment is indicated in the figure legend. The *p* values were expressed as follows: ns, not significant; *, *p*<0.05; **, *p*<0.01; ***, *p*<0.001; and ****, *p*<0.0001.

## Results

### Effect of Pol I inhibitor BMH-21 on RPA12

**Fig 1:**
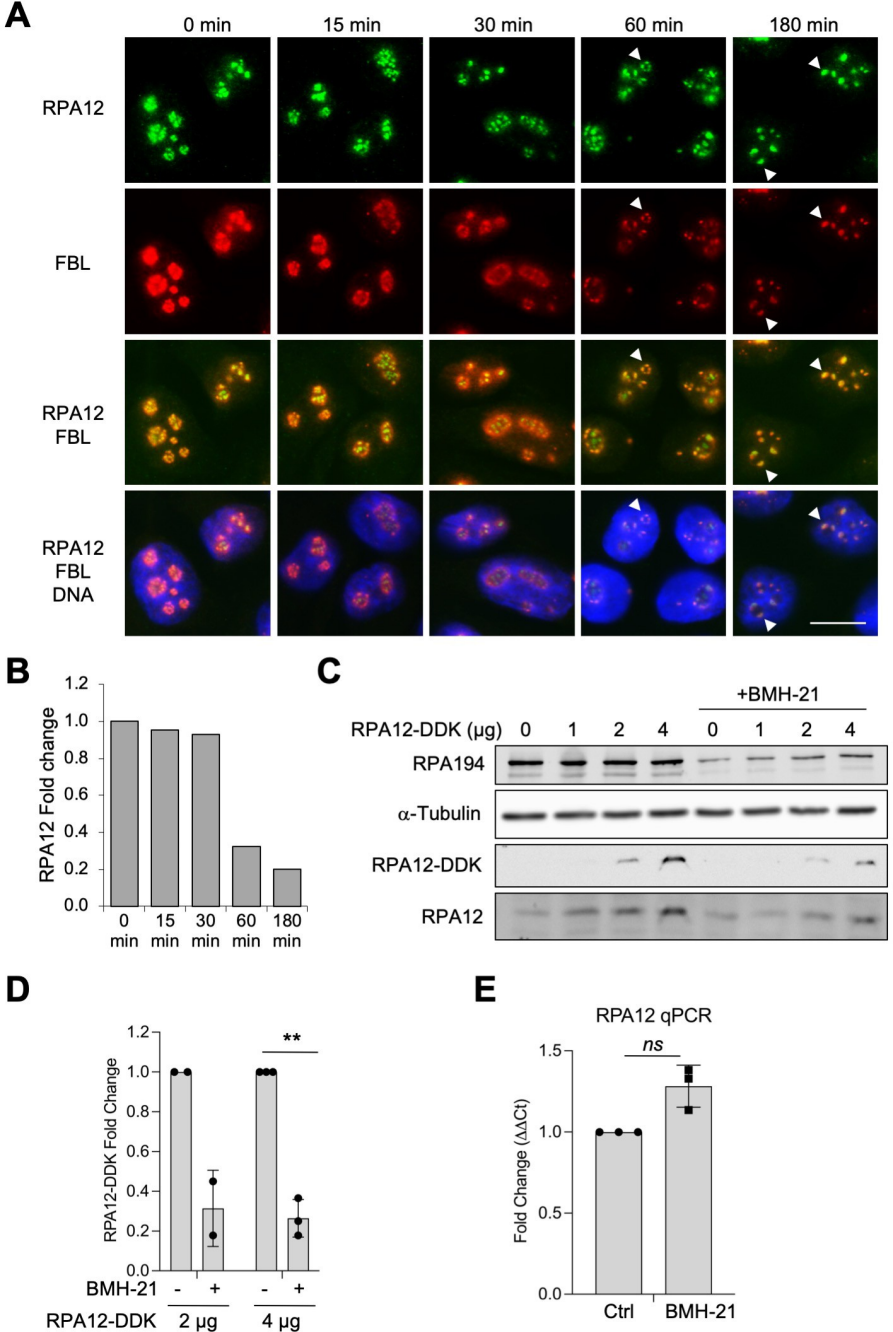
BMH-21 impacts RPA12 nucleolar localization. **(A)** Immunofluorescence staining of A375 melanoma cells treated with BMH-21 for the indicated times. Cells were stained for RPA12 and fibrillarin (FBL). DNA was counterstained using Hoechst. Representative biological replicate of n = 3 is shown. Arrowheads indicate nucleolar cap structures. Scale bar, 10 μm. **(B)** Quantification of the images for RPA12. Fold change is shown. N = 50 cells per treatment. **(C, D)** Ectopic expression of RPA12-DDK. RPA12-DDK expression vector was transfected into A375 cells at the indicated amounts (μg) and treated with BMH-21 (1 μM) for three hours. Cell lysates (30 μg) were analyzed with antibodies for RPA194, DDK and RPA12. **(D)** Quantification of RPA12-DDK in (C). N = 2–3 replicates. Mean fold change ±SD is shown. Student’s two-tailed t-test **, p = 0.00545; *ns*, non-significant. **(E)** qPCR analysis of RPA12 transcript in A375 melanoma cells treated with BMH-21 for 3 hours. Mean fold change ±SD of n = 3 biological replicates is shown. Statistical analysis was conducted using Student’s two-tailed t test. ns, non-significant.

RPA12, the small 13.9 kDa subunit specific for Pol I, is required for the cleavage of nascent rRNA and assists in polymerase backtracking and proofreading [20, 26, 27]. Based on detailed dynamic studies *in vitro*, RPA12.2, the yeast RPA12 homologue, affects nucleotide addition kinetics and elongation complex stability [19, 20]. We have earlier shown that BMH-21, a small-molecule Pol I inhibitor, destabilizes RPA194 [12]. However, the effect of BMH-21 on RPA12 has not been analyzed before. We first conducted a kinetic experiment to assess the impact of BMH-21 on RPA12 using immunofluorescence analysis. BMH-21 (1 μM) was applied to the cells for increasing periods of time, up to 180 minutes, followed by staining of the cells for RPA12 (Fig 1A). Pol I transcription stress by BMH-21 leads to nucleolar reorganization, is amply documented [12, 39], and was monitored by staining for fibrillarin (FBL), an RNA methyltransferase and dense fibrillar center protein required for rRNA processing. BMH-21 treatment caused rearrangement and condensation of the nucleolus at 30–60 minutes of treatment. Nucleolar caps became prominent at 180 min. RPA12 was observed on the outer surface of the nucleolar caps at this time, and partially overlapped with FBL that resided innermost in the caps (Fig 1A). These findings are consistent with the location of Pol I complex proteins during transcription stress [39]. A reduction in RPA12 signal was observed at 180 minutes (Fig 1B), indicating either a decrease in its protein expression or its subcellular redistribution. As we experienced a limitation in the RPA12 antibody performance during immunoblotting analysis (S1 Fig), we ectopically expressed RPA12 tagged with DDK and treated the cells with BMH-21. We observed a decrease in RPA12-DKK supporting the notion that RPA12 abundance could be affected by the drug treatment (Fig 1C and 1D). BMH-21 treatment of the cells did not affect the expression of RPA12 transcript (Fig 1E).

### Influence of RPA12 depletion on Pol I subunits

**Fig 2:**
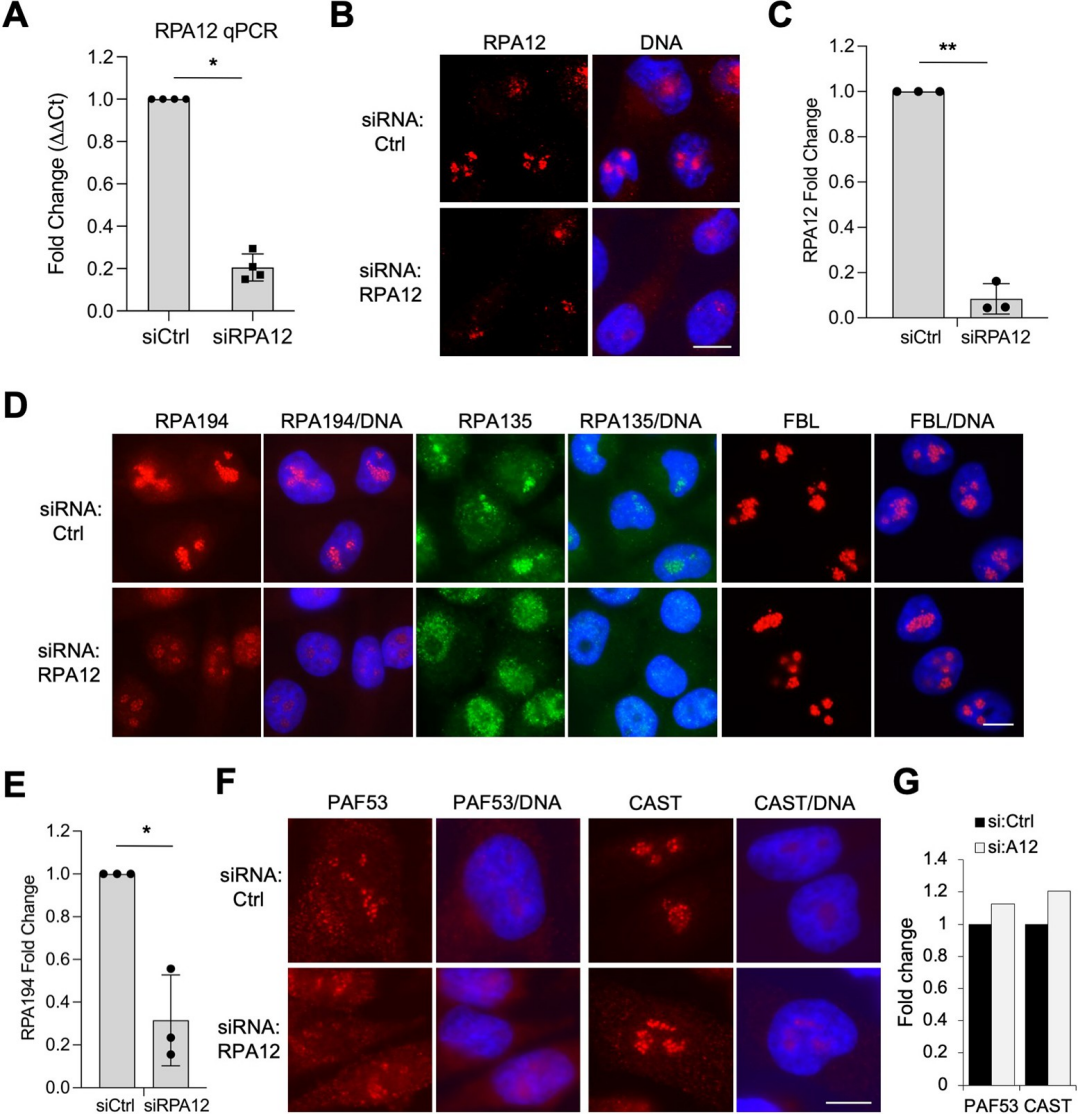
RPA12 knockdown affects the expression and localization of RNA Polymerase I subunits RPA194 and RPA135. **(A) **qPCR analysis of RPA12 transcript in A375 melanoma cells with transient RPA12 knockdown using siRNA. Mean fold change ± SD of n = 4 biological replicates is shown. Mann-Whitney two-tailed t-test *, p < 0.05. **(B)** Immunofluorescence staining of RPA12. Representative biological replicate of n = 4 is shown. **(C)** Quantification of the images for RPA12. Mean fold change ±SD of n = 3 biological replicates is shown. Analysis of N = 50 cells per treatment. Student’s two-tailed t-test **, p = 0.0018. **(D)** Immunofluorescence staining of siCtrl and siRPA12 knockdown cells. A375 melanoma cells were stained for the indicated Pol I subunits and fibrillarin (FBL) following siRNA knockdown of RPA12. DNA was counterstained using Hoechst. Representative biological replicates of n = 4 are shown. **(E)** Quantification of the images for RPA194. Mean fold change ±SD of n = 3 biological replicates is shown. N = 50–100 cells per treatment. Student’s two-tailed t-test *, p = 0.0306. **(F)** Immunofluorescence staining of siCtrl and siRPA12 knockdown cells for PAF53 and CAST. **(G)** Quantification of the images for PAF53 and CAST. N = over 100 cells per treatment. Scale bars, 10 μm.

To investigate the roles of RPA12 in mammalian models, we sought to determine the impact and function of RPA12 in human cells using RNAi against RPA12 using stable lentiviral shRNA knockdown. However, we did not achieve stable, substantive knockdown of RPA12 over serial passaging using RPA12 shRNAs despite the acute knockdown being effective (S2 Fig). Therefore, transient siRNA treatment using multiple siRNAs against RPA12 was used for cellular knockdown and was validated using qPCR (Fig 2A). An 80% reduction in cellular RPA12 expression was achieved, with little variance between biological replicates. We additionally used immunofluorescence analysis to detect RPA12 protein levels, which showed prominent decrease in RPA12 protein expression (Fig 2B and 2C).

We then analyzed the impact of RPA12 knockdown on Pol I subunits using immunofluorescence. Compared to control siRNA-transfected cells (siCtrl), RPA12 knockdown cells displayed a decrease in the signal intensity of RPA194, the largest catalytic subunit of Pol I (Fig 2D and 2E). RPA135, the second largest subunit, showed a shift in localization from the nucleolus to nucleoplasm upon RPA12 knockdown (Fig 2D). Similar changes were not observed for FBL (Fig 2D) or the other Pol I subunits PAF53 and CAST (Fig 2F and 2G). These findings imply RPA12 depletion impacts the enzyme complex in a Pol I subunit-specific manner that does not activate a nucleolar stress response.

### RPA12 and BMH-21 affect the expression and stability of Pol I subunit RPA194

**Fig 3:**
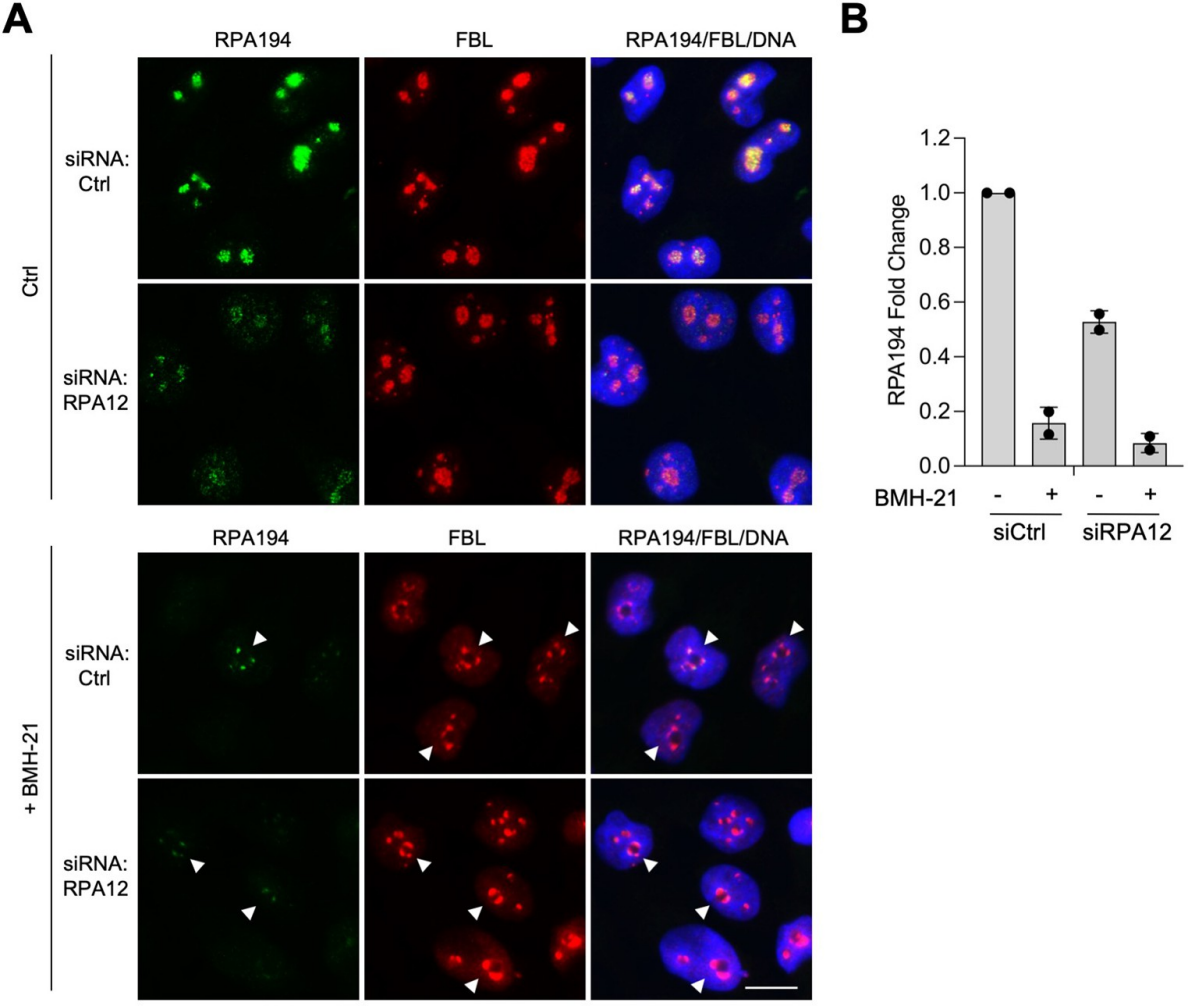
Depletion of RPA12 does not modify the effect of BMH-21 on RPA194. **(A)** Immunofluorescence staining of siCtrl and RPA12 knockdown cells. A375 melanoma cells were stained for RPA194 and FBL following siRNA knockdown of RPA12. DNA was counterstained using Hoechst. Cells were treated with vehicle (Ctrl) or BMH-21 (1 μM) for 3 hours. Representative biological replicate of n = 4 is shown. Scale bar, 10 μm. **(B)** Quantification of the images for RPA194. Fold change is shown. N = 2 biological replicates and 50–150 cells per treatment.

Next, we asked whether the Pol I inhibitor-induced destabilization of RPA194 is dependent on RPA12. We treated control siRNA-transfected and RPA12 knockdown cells with BMH-21, followed by immunofluorescence staining for RPA194 and FBL as control (Fig 3A). As in Fig 2D and 2E, the signal for RPA194 decreased in the RPA12 knockdown cells (Fig 3B). As previously documented [3, 12], BMH-21 treatment led to a robust decrease in RPA194 nucleolar staining in both the control and RPA12 depleted cells (Fig 3A and 3B).

**Fig 4:**
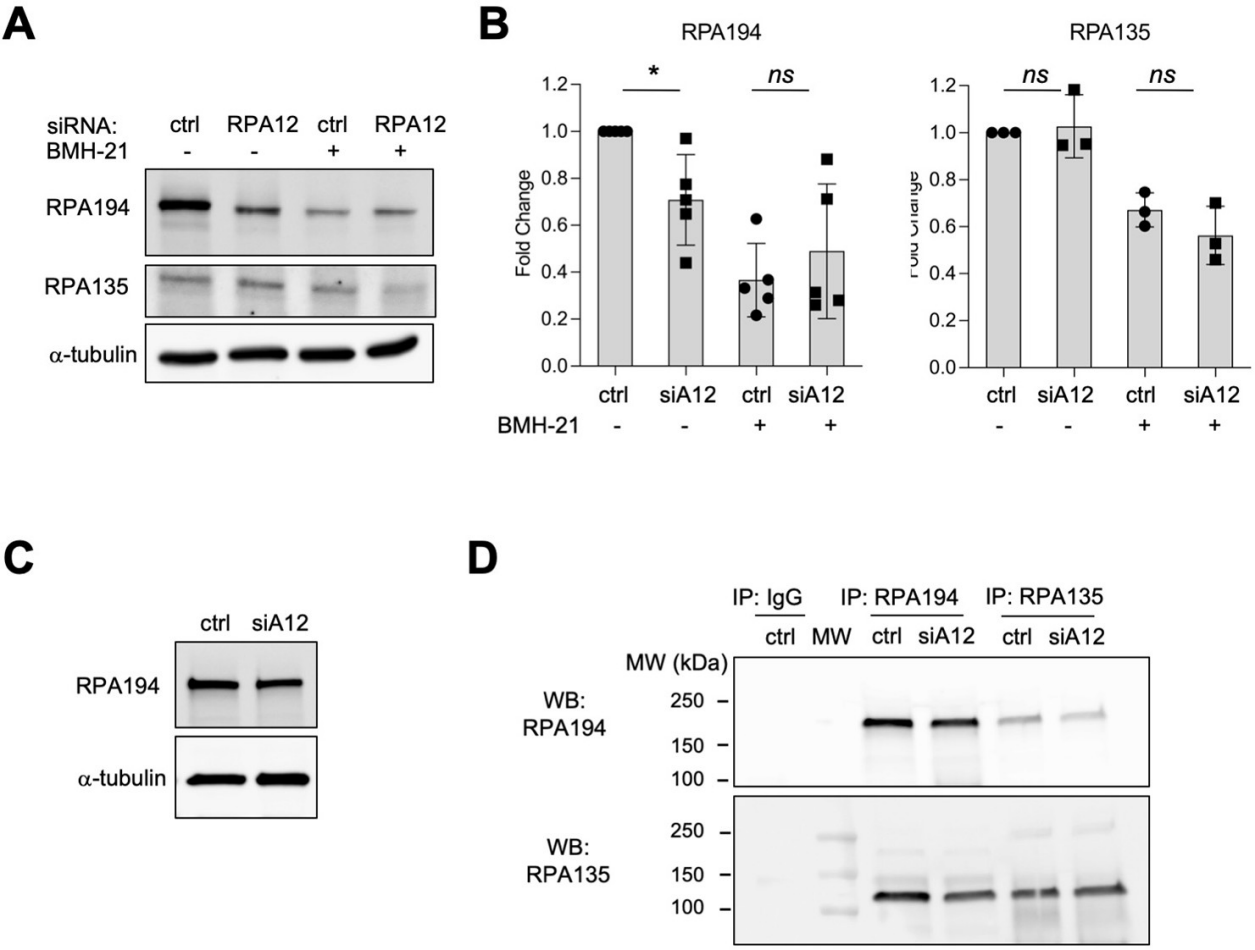
Influence of RPA12 on RPA194 and RPA135 proteins and their interaction. Ctrl siRNA and RPA12 siRNA transfected A375 melanoma cells were treated with BMH-21 (1 μM) for 3 hours. **(A)** Western blot analysis. Cell lysates (20 μg/lane) were immunoblotted for RPA194 and RPA135. **(B)** Quantification for RPA194 of n = 5 biological replicates and RPA135 of n = 3 biological replicates. Data are represented as mean ± SD. Statistical analysis was conducted using non-parametric Mann-Whitney two-tailed t test. ns, non-significant. *, *p*<0.05. **(C and D)** Coimmunoprecipitation analyses. Cell lysates **(C)** were immunoprecipitated with RPA194 and RPA135 antibodies or control IgG followed by immunoblotting **(D)** for RPA194 and RPA135 as indicated. Molecular weight markers are shown to the left.

We then analyzed whether RPA12 affects RPA194 steady-state level by western blotting. As shown by a representative experiment, and quantification of multiple biological replicates, RPA12 knockdown led to a significant 30% decrease in RPA194 protein expression (Fig 4A and 4B). However, there was no change in RPA135 protein by RPA12 knockdown (Fig 4A and 4B). Further assessment into the effect of either RPA12 knockdown, treatment with BMH-21, or their combination on the transcripts of RPA194 or RPA135 by qPCR did not reveal any change, indicating that the changes observed in RPA194 protein are likely posttranscriptional (S3A and S3B Fig).

We next analyzed the dependency of RPA194 turnover by BMH-21 on RPA12. Consistent with our previous publication [12], BMH-21 caused a prominent decrease of RPA194 in cells transfected with control siRNA and this decrease was not significantly altered by the knockdown of RPA12 (Fig 4A and 4B). We observed a decrease in RPA135 by the drug treatment; however, RPA12 knockdown did not modify this response (Fig 4A and 4B). Thus, we conclude that while RPA12 affects the basal stability of RPA194, the drug-induced turnover of RPA194 is independent of RPA12 expression.

In the yeast, A190 (corresponding to human RPA194) and A135 (corresponding to human RPA135) form a stable stochiometric complex [40]. Since we observed that RPA12 knockdown affected RPA194 protein expression and RPA135 localization we questioned whether these alterations could cause complex dissociation and the loss of enzymatic activity upon RPA12 knockdown. We therefore performed a Co-IP experiment to determine the effect of RPA12 on their interaction and used either RPA194 or RPA135 antibodies for the pulldowns followed by reciprocal immunoblotting for RPA194 and RPA135 (Fig 4C and 4D). The co-precipitation of either RPA194 or RPA135 was independent of RPA12 knockdown (Fig 4D). These findings indicate that the RPA194:RPA135 complex does not dissociate upon RPA12 knockdown.

### RPA12 depletion does not affect Pol I transcription activity or Pol I occupancy on rDNA

**Fig 5:**
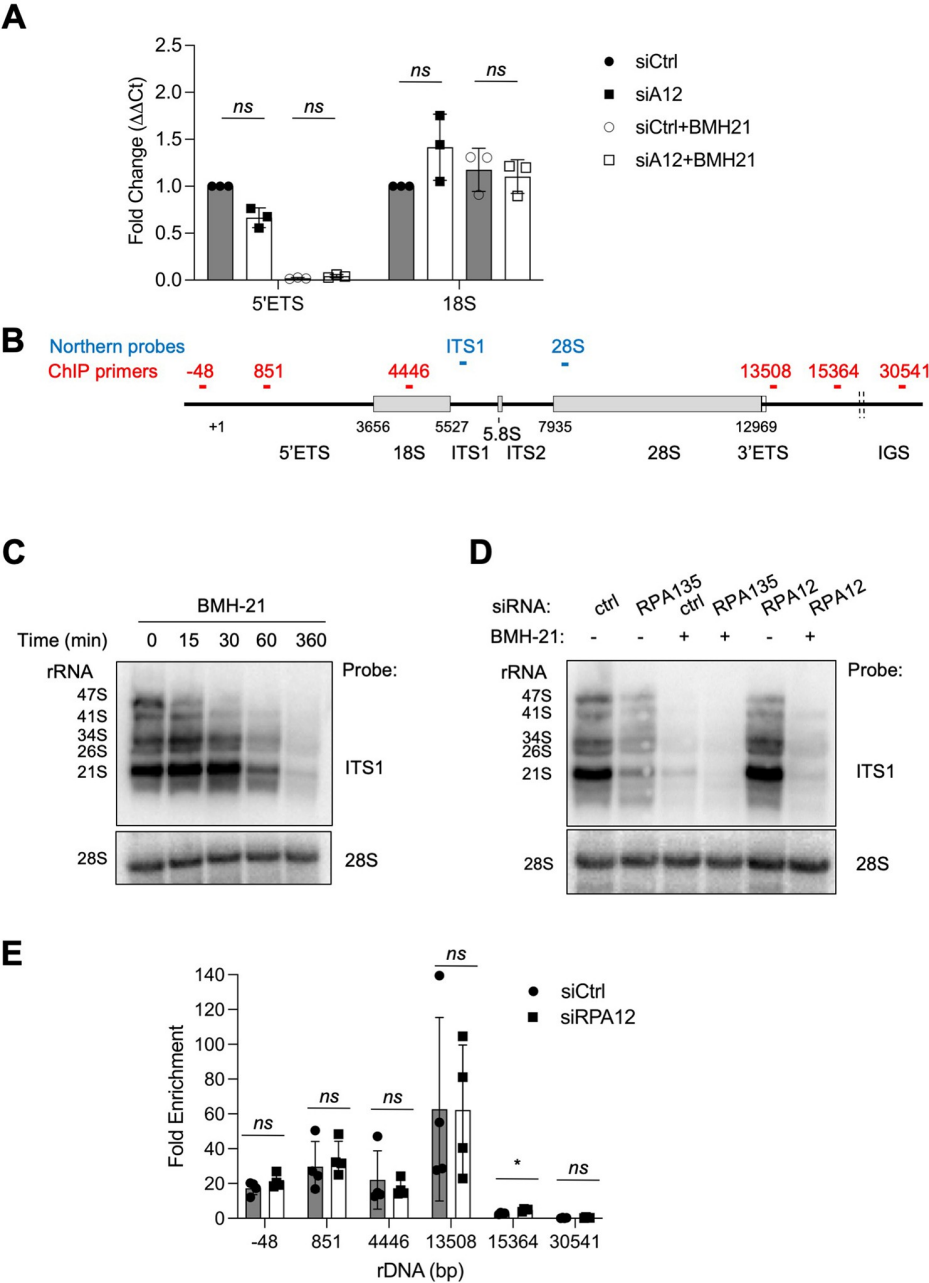
RPA12 is not essential for Pol I transcription, nor the enzyme chromatin occupancy. **(A)** qPCR analysis of rRNA synthesis following transfection with siCtrl and RPA12 siRNAs in A375 melanoma cells. Cells were treated with and without BMH-21 (1 μM) for 6 hours. Primer pairs for 5’ETS and 18S rRNAs were used. Fold change for n = 3 biological replicates are shown, and data are represented as mean ± SD. Statistical analysis was conducted using non-parametric Mann-Whitney two-tailed test. ns, non-significant. **(B)** Schematic outline for the rRNA coding region and the probes used for Northern analysis (*blue*) and primers used for ChIP (*red*). **(C and D)** Northern blot analyses. **(C)** A375 cells were treated with BMH-21 for the indicated times and total RNA was prepared. 6 μg RNA/lane was loaded. **(D)** Total RNA was isolated from A375 melanoma cells transfected with siCtrl, RPA12 or RPA135 siRNAs and treated with or without BMH-21 for 6 hours. RNA (0.9 μg per lane) was loaded and the blots were probed with ITS1 and 28S probes as indicated. rRNA processing transcripts are indicated to the left. **(E)** Chromatin immunoprecipitation analysis following siCtrl and RPA12 knockdown in A375 melanoma cells. Primer pairs for the promoter (-48), 5’ETS (+851), 18S (+4446), two termination sites (+13508, +15364) and non-coding IGS (+30541) regions were used. Fold enrichment of n = 4 biological replicates is shown. Data are represented as mean ± SD. Statistical analysis was conducted using non-parametric Mann-Whitney two-tailed test. ns, non-significant.

To further investigate whether Pol I activity is impacted by knockdown of RPA12 we analyzed the expression of the 47S precursor rRNA transcript by qPCR using primers specific to the short-lived 5’ETS rRNA and the stable mature 18S rRNA (Fig 5A). RPA12 knockdown did not affect the expression of the 5’ETS or 18S rRNAs. As expected, BMH-21 treatment robustly decreased expression of the 5’ETS rRNA, while it had no effect on the 18S rRNA (Fig 5A). RPA12 knockdown did not alter these drug-induced responses.

We wanted to further assess this outcome using Northern blotting. We used an internal transcribed spacer 1 (ITS1) probe specific to the precursor rRNA and a 28S probe specific to the mature rRNA, which also served as loading control (Fig 5B). First, we evaluated the temporal response to BMH-21. We observed a rapid reduction of the 47S precursor rRNA within 15 minutes, and a complete loss of the 21S, 26S, 34S, 41S rRNA processing intermediates by 6 hours (Figs 5C and S4A). Then we evaluated the effect of RPA12 knockdown on the rRNA synthesis and included as a control the knockdown of RPA135 (S4B and S4C Fig). While RPA135 knockdown prominently abrogated rRNA synthesis, RPA12 knockdown did not affect the processing intermediates and had only a minor effect on the 47S precursor rRNA (Figs 5D and S4D). BMH-21 abrogated rRNA synthesis in both the RPA12 and RPA135 knockdown cells (Fig 5D).

Lastly, we conducted chromatin immunoprecipitation (ChIP) analysis to assess whether RPA12 affects Pol I occupancy on the rRNA gene. ChIP was conducted on RPA12 knockdown and siCtrl cells using primers for the coding region (promoter, 5’ETS, 18S), two termination sites (T1, T2) and the non-coding intragenic spacer (IGS). There was no major difference in the occupancy of RPA194 on the rRNA gene locus upon RPA12 knockdown (Fig 5E).

**Fig 6:**
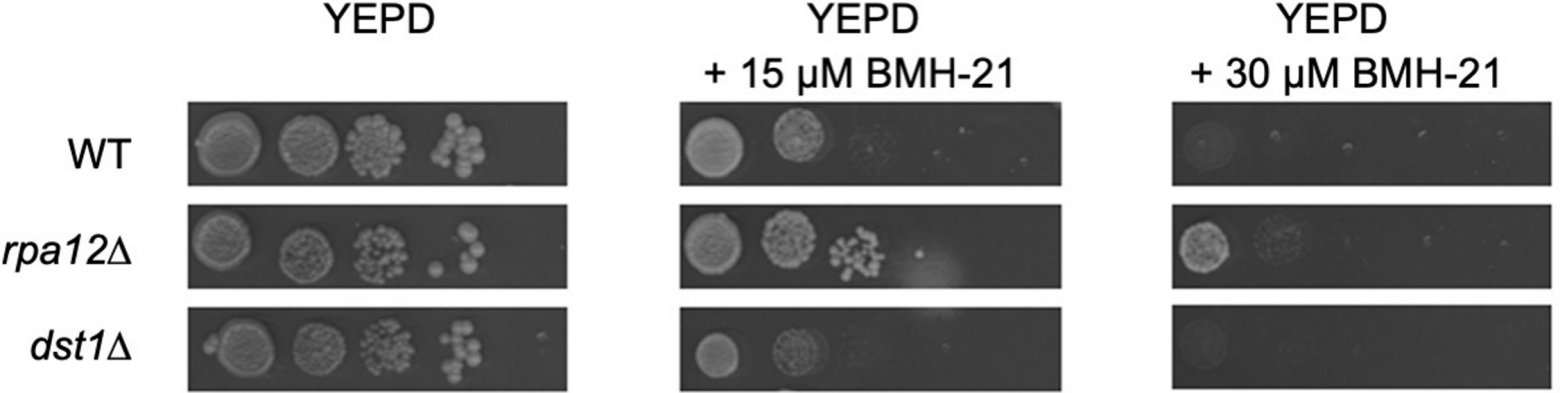
Deletion of *RPA12* renders yeast cells partially resistant to BMH-21. Ten-fold serial dilutions of WT, *rpa12*.*2*Δ, and *dst1*Δ strains were spotted onto YEPD plates with indicated concentrations of BMH-21. Plates were incubated for six days at 23°C.

We previously demonstrated that the effects of BMH-21 on rDNA transcription are conserved across eukaryotic species, including yeast [14, 15, 17]. To determine whether complete deletion of *RPA12*.*2* influences cellular responses to BMH-21, we plated WT, *rpa12*.*2*Δ, and *dst1*Δ cells on YEPD and grew cells for six days at 23°C (Fig 6). *DST1* encodes the transcription factor TFIIS. Both RPA12.2 and TFIIS influence nascent RNA cleavage and serve similar roles for Pols I and II respectively. Thus, *dst1*Δ was included as a negative control.

Interestingly, *rpa12*.*2*Δ cells formed colonies even in the presence of 30 μM BMH-21, whereas WT and *dst1*Δ cells were less viable in the presence of BMH-21 and colonies that did form were smaller, indicating slower cell proliferation (Fig 6). These data show that the presence of RPA12 renders Pol I more sensitive to inhibition by BMH-21. The simplest interpretation of this observation is that Pol I forms a less stable transcription elongation complex when RPA12 is expressed [20]. Deletion of *RPA12*.*2* results in more stable transcription elongation complexes and reduced transcription elongation rate by Pol I. These properties may render Pol I more resistant to the transcriptional stress induced by BMH-21, much like Pol II.

## Discussion

RPA12 is bestowed with several key activities in Pol I transcription. These include nucleotide addition, RNA cleavage, enzyme backtracking and proofreading, and transcription termination [18, 20, 26, 27, 41]. Not surprisingly, *RPA12*.*2* deletion has temperature-associated lethality in yeast. However, when grown at cold temperatures, or if only the C-terminus containing the TFIIS paralog domain is deleted, viability is sustained [24, 42]. We were unsuccessful in establishing long-term stable knockout of RPA12 using shRNA or sgRNAs (not shown) in mammalian cancer cells, while transient knockdown was achieved using both shRNA and siRNA. This suggests that RPA12 knockdown does not immediately compromise mammalian cell proliferation; however, the sustained, long-term depletion of RPA12 leads to loss of cell viability. Further, we show that knockdown of RPA12 in mammalian cells leads to decreased expression of Pol I catalytic subunit RPA194. Despite this, Pol I gene occupancy, transcription, and rRNA synthesis remain unaffected in these short-term experiments. We infer that cancer cells tolerate transient fluctuations in the expression of their large subunits and maintain stable association with the gene throughout transcription cycles possibly due to their abundant expression.

The small-molecule Pol I inhibitor BMH-21 targets Pol I via intercalation of the GC-rich rDNA leading to inhibition of transcription initiation and elongation, ultimately leading to the degradation of Pol I subunit RPA194 [12, 14–17]. Given that the elongation blocks require enzyme backtracking and RNA cleavage for resolution we analyzed the effect of this Pol I inhibitor on RPA12. Following application of the inhibitor onto cells, RPA12 was detected within an hour in nucleolar cap structures and over time its expression in the nucleolus was reduced. Our data show a reduction in ectopically expressed RPA12 suggesting that also its turnover could be affected by BMH-21. However, we are unable to confirm this finding on the endogenous protein due to the limitation in the performance of the antibody in western blotting analyses. This prompted our investigation into whether RPA12 knockdown influences the stability of Pol I subunits, either at steady-state or in response to Pol I inhibitor, and their ability to form a stable core complex.

We analyzed the expression of Pol I subunits, specifically the two largest subunits RPA194 and RPA135 following depletion of RPA12. We observed a decrease in RPA194 protein and a shift of RPA135 from the nucleolus into the nucleoplasm. The decrease in RPA194 was specific to this subunit, since RPA12 knockdown had negligible impact on RPA135 steady-state level. This is interesting as the stoichiometric balance of human Pol I shown by structural reconstructions denote one RPA194 subunit to one RPA135 subunit [43]. Hence it is possible that instability or delocalization of one subunit could disrupt the binding or structure of Pol I. However, Co-IP studies indicate that despite the decrease in RPA194 expression and relocalization of RPA135 there is no dissociation of the core complex formed by RPA194 and RPA135 upon RPA12 knockdown. The findings reported here are consistent with those in yeast as Nogi et al. found that A190, the RPA194 yeast homologue, had decreased expression in *RPA12*.*2* deletion strain [42] and that RPA12 is involved in the recruitment and docking of the catalytic core to the Pol I complex [18, 24, 42]. Thus, our data supports these findings by indicating a role for RPA12 in human Pol I stability.

Even if the structure and core complex of Pol I is not disrupted by RPA12 knockdown we postulated RPA12 knockdown could cause functional changes that negatively affect the transcriptional capacity of Pol I. To determine whether RPA12 knockdown has an impact on Pol I transcription, RNA was isolated from RPA12 knockdown cells and the mature and intermediate transcriptional products were analyzed. There was no major change in Pol I transcription following RPA12 knockdown of the short-lived 5’ ETS transcript, mature 18S or 28S rRNAs, or their intermediate processed forms as measured by qPCR and Northern hybridization. Given this, the decrease in RPA194 expression or alteration of RPA135 location upon RPA12 knockdown does not impair Pol I transcription, whereas RPA135 knockdown substantially did. Therefore, the relative levels of RPA135 and RPA194 which remain in the nucleolus upon RPA12 knockdown must be sufficient for Pol I to continue at a rate equivalent to normal basal conditions.

Due to the RPA12 cleavage activity, we hypothesized that RPA12 knockdown could affect the termination of Pol I by causing the polymerase to stall without the ability to release the nascent RNA. However, based on ChIP data there are no alterations in Pol I occupancy throughout the coding region or termination sites on the rDNA template. Using NET-seq, Clarke et al. showed that deletion of RPA12.2 in yeast led to read-through capability of the polymerase enabling it to bypass T1 and T2 termination sites to ultimately engage in termination at a later promoter—proximal Reb1 binding site [41]. These findings support earlier studies by Prescott et al. which highlighted the homology between RPA12.2, Rpb9, and C11 in the termination of yeast RNA polymerases I, II and III, respectively, and showed a loss of polymerase termination in RPA12.2 deleted yeast with readthrough transcription into the spacer region [18]. It remains a possibility that the human Pol I utilizes a similar mechanism.

## Conclusion

Here we showed that the transient depletion of RPA12, a core component of the Pol I enzyme complex and an essential factor for RNA cleavage, led to a decreased steady-state protein expression of the catalytic subunit RPA194. Despite this, RPA12 is nonessential for continued rRNA synthesis and chromatin engagement of the polymerase in human cancer cells. However, long-term sustained depletion of RPA12 was not achieved. This study has the limitation that it was performed in only one cancer cell line. Additional studies will be needed to investigate the implications of RPA12 knockdown and the functional impact it has on Pol I enzyme composition, transcription, and termination. As yeast RPA12.2 conveys proofreading functions for the rRNA transcript and its depletion leads to high polymerase error rates, it is plausible that long-term ablation of its activity leads to rRNA transcription errors that compromise ribosome function and cell survival.

## Supporting information

S1 Fig RPA12 antibody performs poorly in detection of endogenous RPA12 in A375 melanoma cells. A375 melanoma were transfected with siRNAs against RPA12 and cell lysates were prepared after 72 hours. Cell lysates (30 μg/lane) were loaded in a 4–20% gradient gel and probed with 1:100 dilution of anti-RPA12 antibody (D10). Non-specific higher molecular weight bands were detected, whereas the detection of bands at 14 kDa expected for RPA12 was unreliable. Thereafter the membranes were probed for RPA194 (195 kDa) and histone H3 (15 kDa) as controls.
(TIF)

S2 Fig Transient and stable shRNA knockdown of RPA12. qPCR analysis of RPA12 in A375 melanoma cells with RPA12 knockdown following lentiviral shRNA transduction. **(A)** Knockdown efficiency following a 72-hour incubation. Four independent shRNAs were evaluated and results are shown for the shRNA (pLKO-shRNA-ZNRD1-19074) with the most effective knockdown. **(B)** Stable cells transduced with pLKO-shRNA-ZNRD1-19074 lentivirus were selected using puromycin and propagated over serial passaging. Passage numbers are indicated below.
(TIF)

S3 Fig RPA12 knockdown does not affect RPA194 or RPA135 transcripts. A375 melanoma cells were transfected with siCtrl, RPA12 or RPA135 siRNAs and treated with or without BMH-21 for 3 hours. qPCR analyses were conducted for **(A)** RPA194 and **(B)** RPA135 transcripts. N = 4 biological replicates, mean ±SD is shown. Student’s two-tailed t-test, *ns*, non-significant.
(TIF)

S4 Fig Knockdown efficiency for RPA12 and RPA135 using siRNA. **(A)** qPCR quantification for 5’ETS precursor rRNA using RNA samples prepared for Northern blotting in Fig 5C. **(B-D)** qPCR quantification using RNA samples prepared for Northern blotting in Fig 5D. Expression of **(B)** RPA12, **(C)** RPA135 and **(D)** 5’ETS precursor rRNA.
(TIF)

S1 Raw images (PDF)

## Abbreviations

Pol I: RNA polymerase I
rRNA: ribosomal RNA
rDNA: ribosomal DNA
IF: Immunofluorescence
qPCR: quantitative polymerase chain reaction
Co-IP: co-immunoprecipitation
ChIP: chromatin immunoprecipitation
