# Supplementary figures and images for: Expression of RNA polymerase I catalytic core is influenced by RPA12
Source: PLoS One. 2023 May 11;18(5):e0285660. doi: 10.1371/journal.pone.0285660 (PMC10174586; doi:10.1371/journal.pone.0285660)

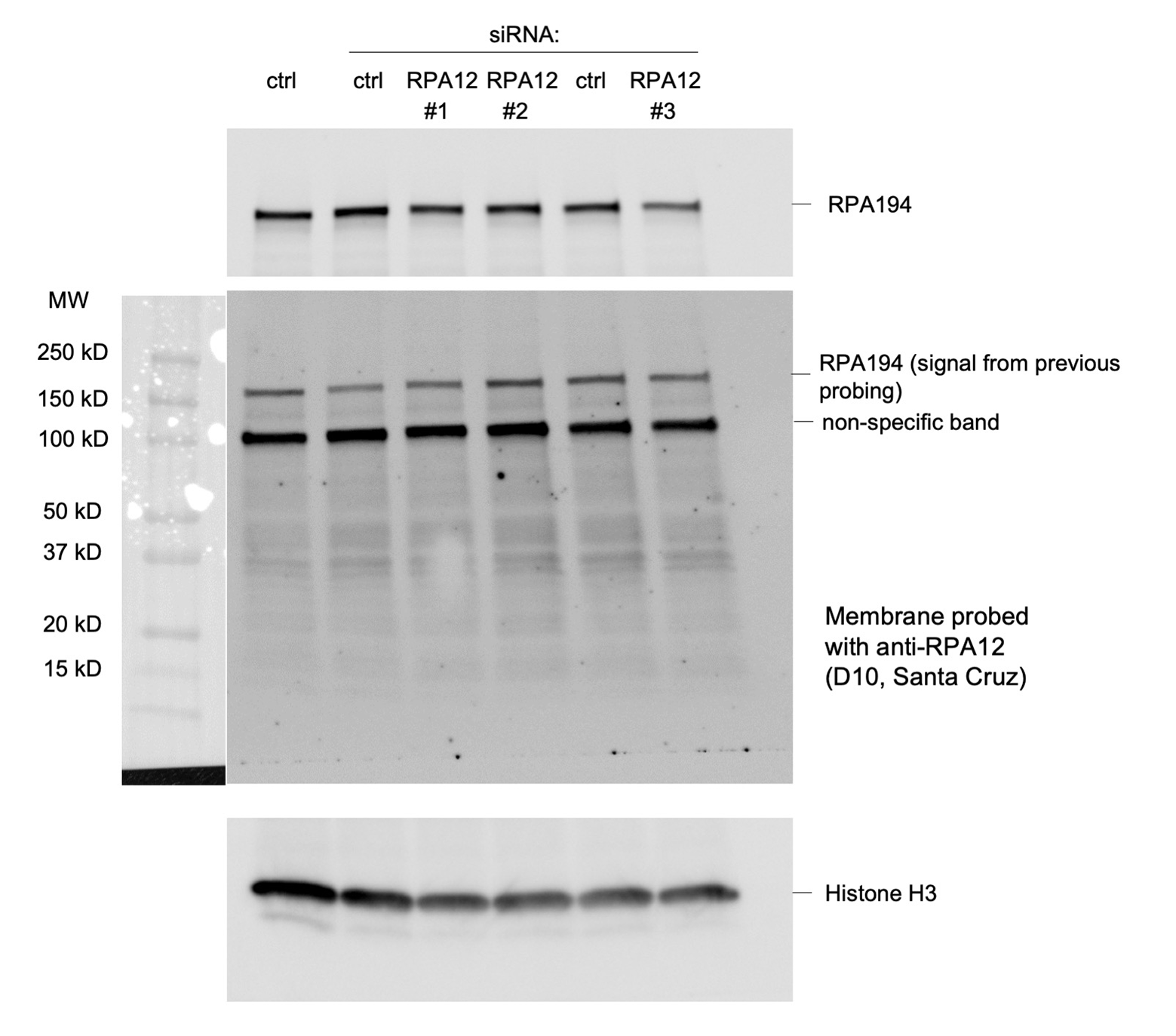

Supplement: S1 Fig — A375 melanoma were transfected with siRNAs against RPA12 and cell lysates were prepared after 72 hours. Cell lysates (30 μg/lane) were loaded in a 4–20% gradient gel and probed with 1:100 dilution of anti-RPA12 antibody (D10). Non-specific higher molecular weight bands were detected, whereas the detection of bands at 14 kDa expected for RPA12 was unreliable. Thereafter the membranes were probed for RPA194 (195 kDa) and histone H3 (15 kDa) as controls. (TIF) [file pone.0285660.s001.tif]

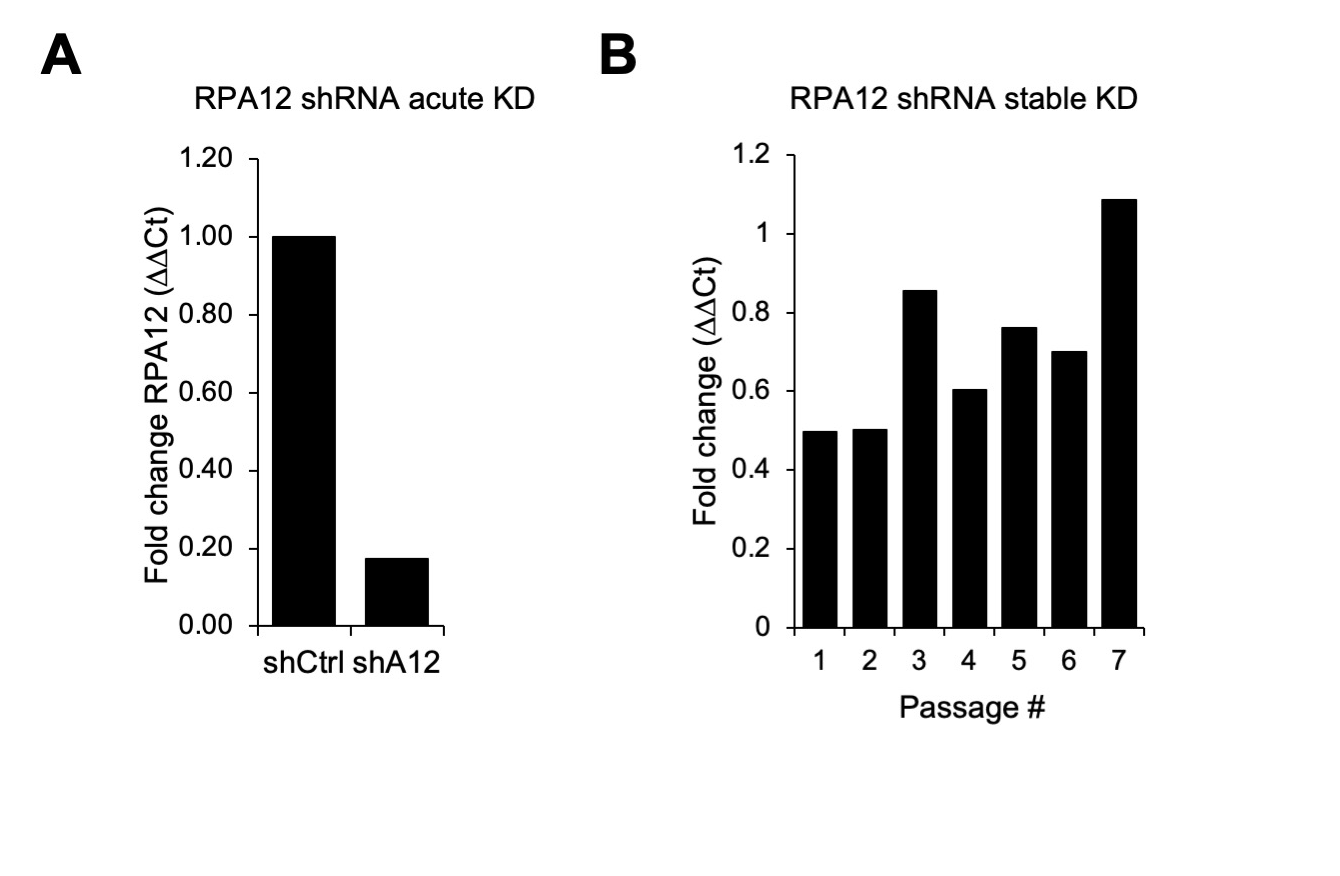

Supplement: S2 Fig — qPCR analysis of RPA12 in A375 melanoma cells with RPA12 knockdown following lentiviral shRNA transduction. (A) Knockdown efficiency following a 72-hour incubation. Four independent shRNAs were evaluated and results are shown for the shRNA (pLKO-shRNA-ZNRD1-19074) with the most effective knockdown. (B) Stable cells transduced with pLKO-shRNA-ZNRD1-19074 lentivirus were selected using puromycin and propagated over serial passaging. Passage numbers are indicated below. (TIF) [file pone.0285660.s002.tif]

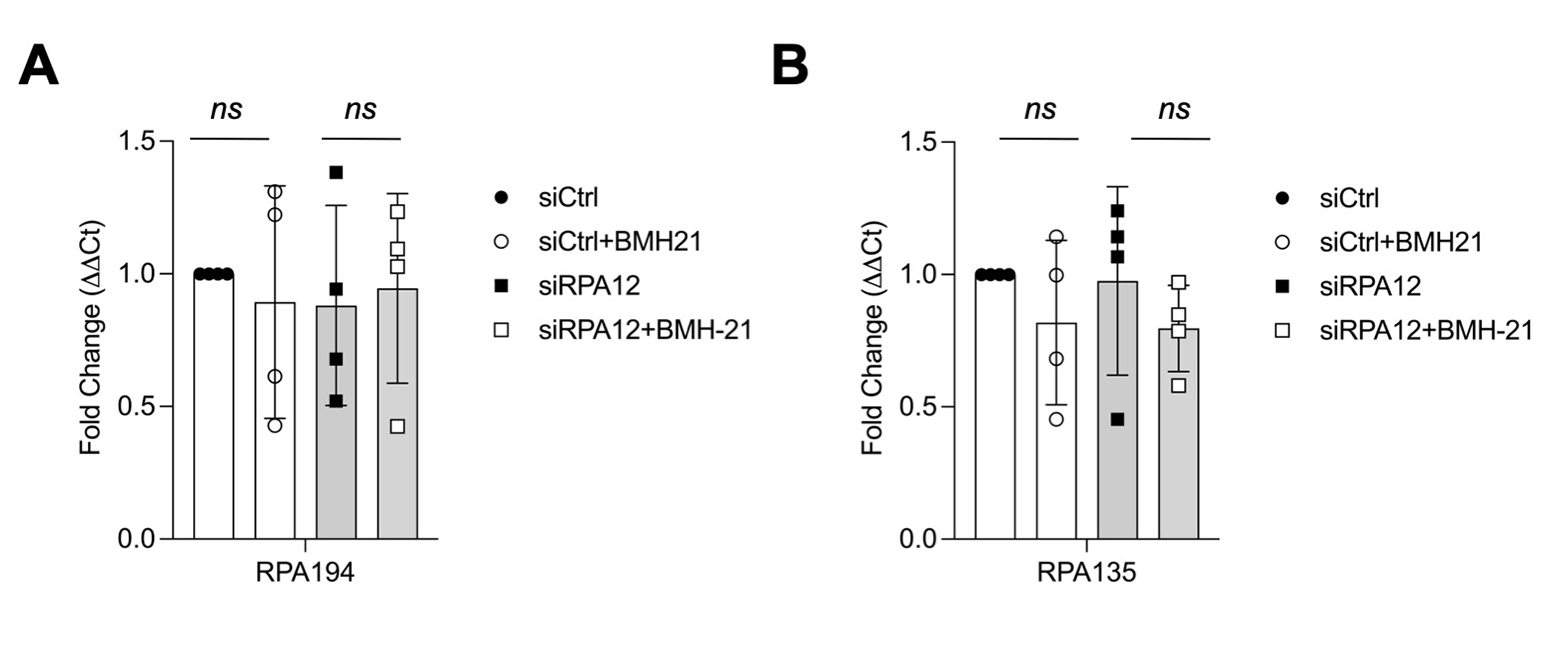

Supplement: S3 Fig — A375 melanoma cells were transfected with siCtrl, RPA12 or RPA135 siRNAs and treated with or without BMH-21 for 3 hours. qPCR analyses were conducted for (A) RPA194 and (B) RPA135 transcripts. N = 4 biological replicates, mean ±SD is shown. Student’s two-tailed t-test, ns, non-significant. (TIF) [file pone.0285660.s003.tif]

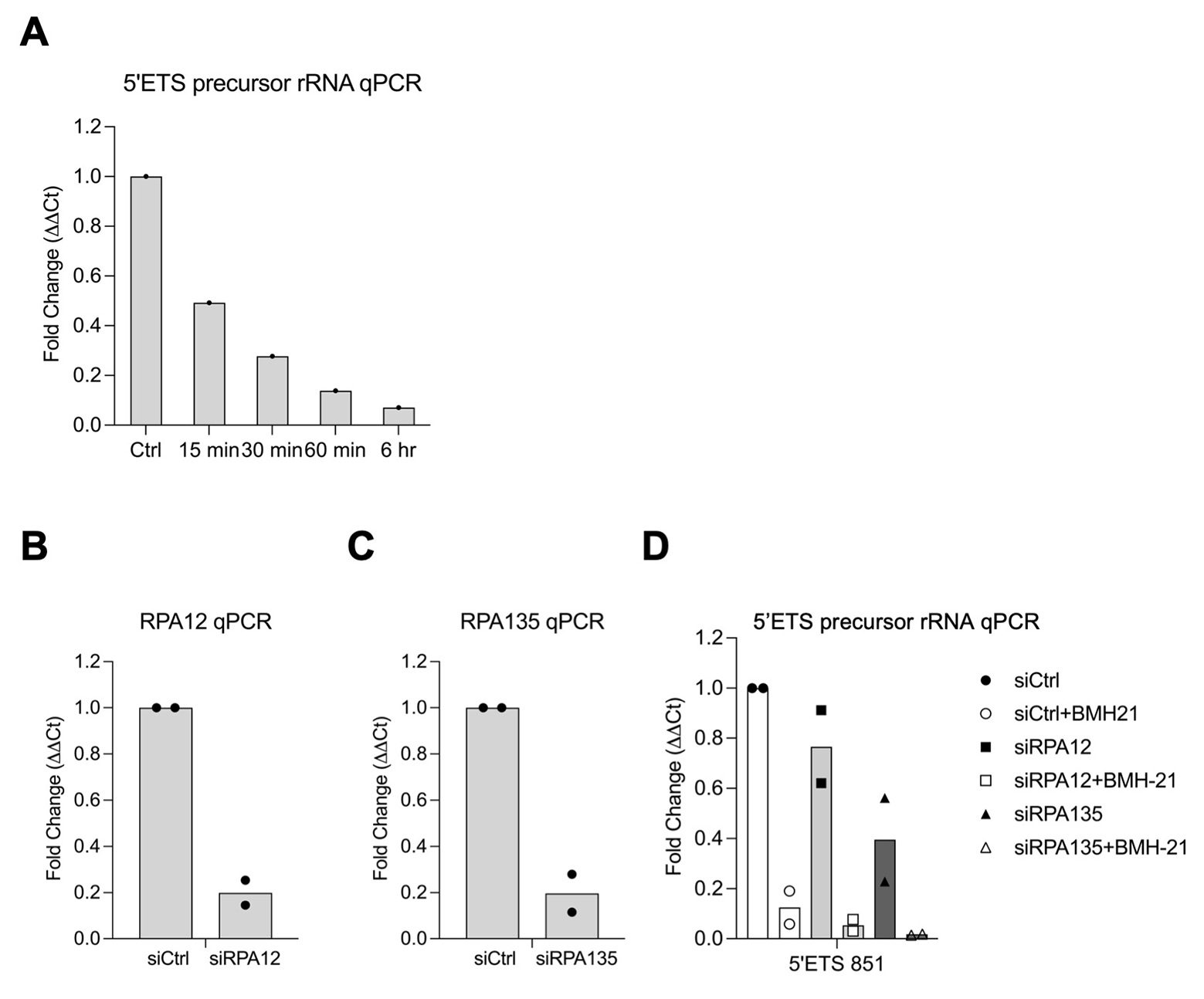

Supplement: S4 Fig — (A) qPCR quantification for 5’ETS precursor rRNA using RNA samples prepared for Northern blotting in Fig 5C. (B-D) qPCR quantification using RNA samples prepared for Northern blotting in Fig 5D. Expression of (B) RPA12, (C) RPA135 and (D) 5’ETS precursor rRNA. (TIF) [file pone.0285660.s004.tif]
