# Supplementary material for: Expression of RNA polymerase I catalytic core is influenced by RPA12
Source: PLoS One. 2023 May 11;18(5):e0285660. doi: 10.1371/journal.pone.0285660 (PMC10174586; doi:10.1371/journal.pone.0285660)

Uncropped Fig. 1D

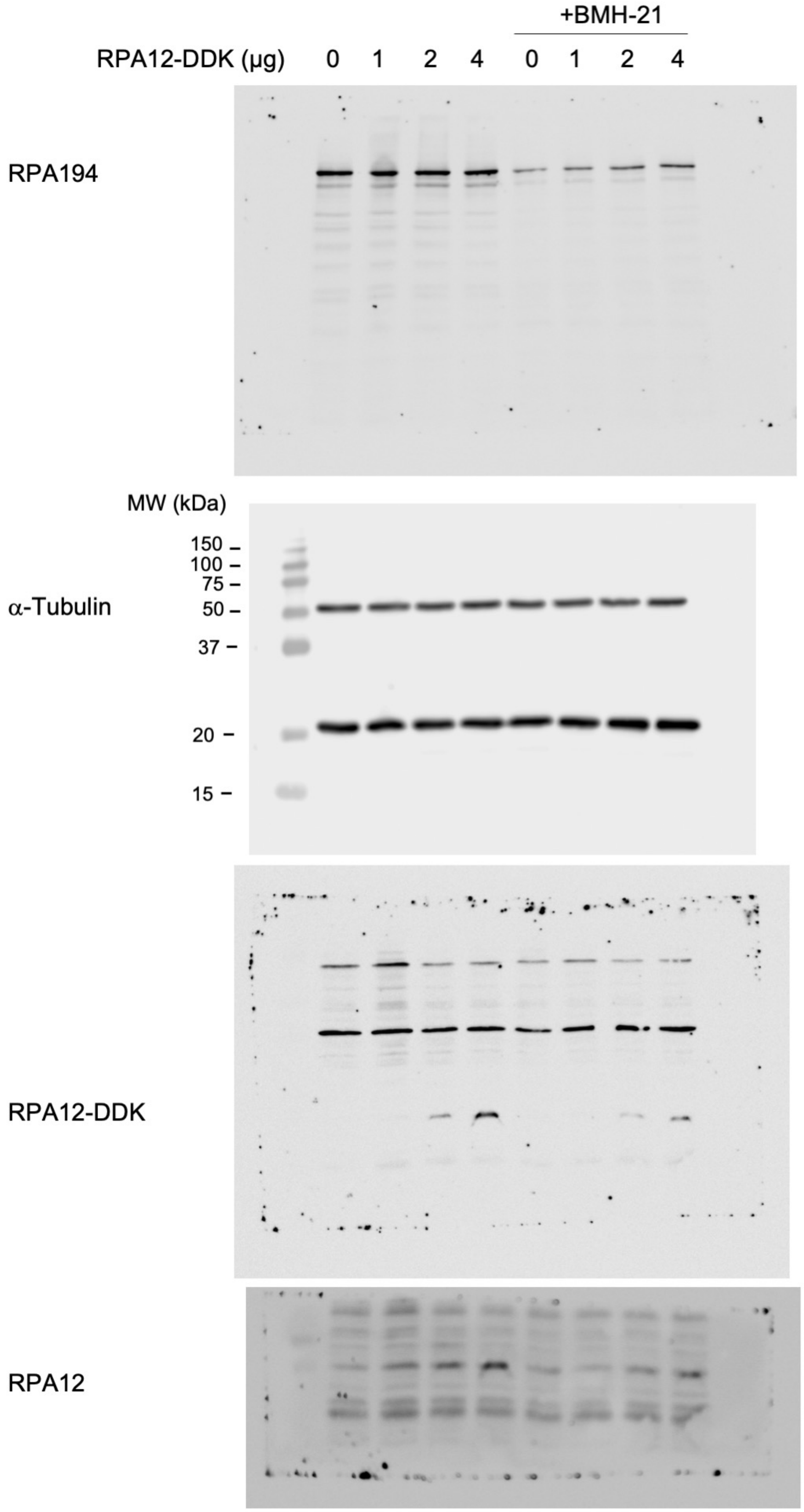

Uncropped Fig. 4A

RPA194 (top panel) RPA135 (middle panel) α-tubulin (bottom panel)

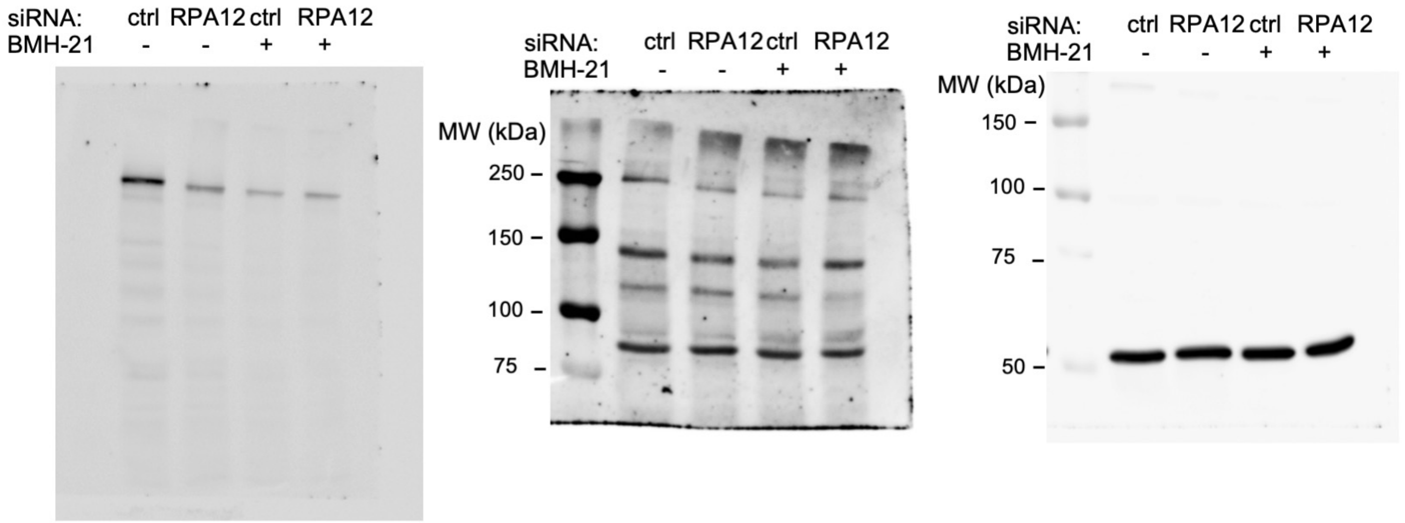

Uncropped Fig. 4C

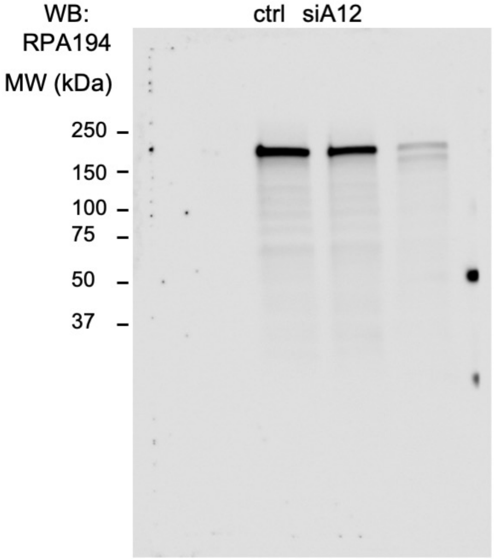

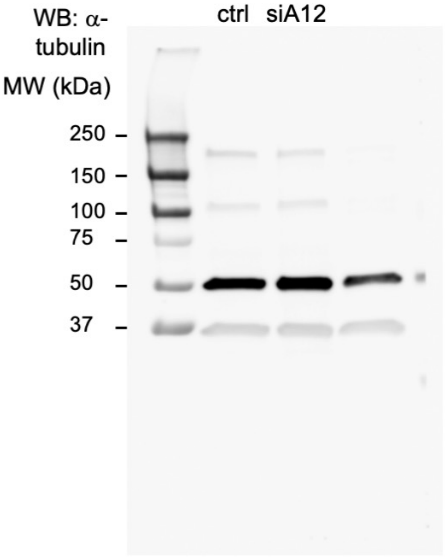

Uncropped Fig. 4D

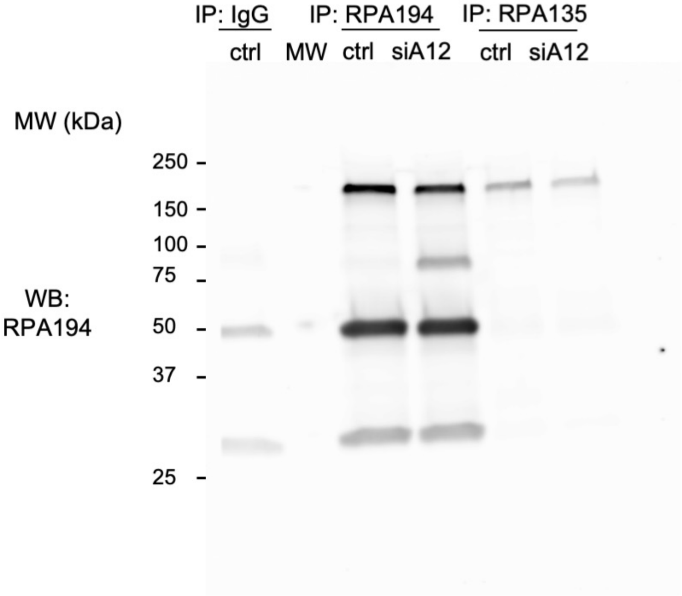

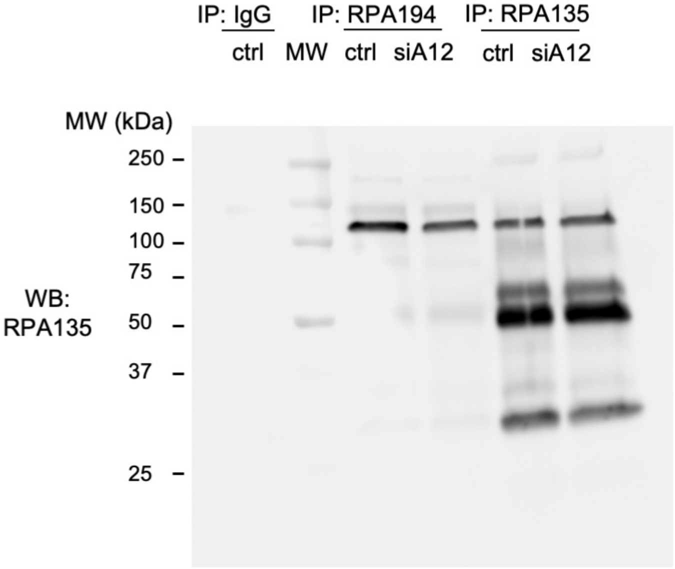

Uncropped 5C ITS1

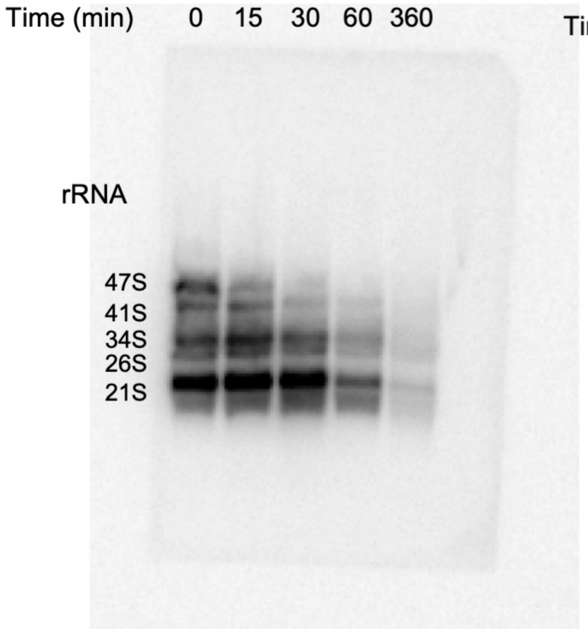

Uncropped 5C 28S

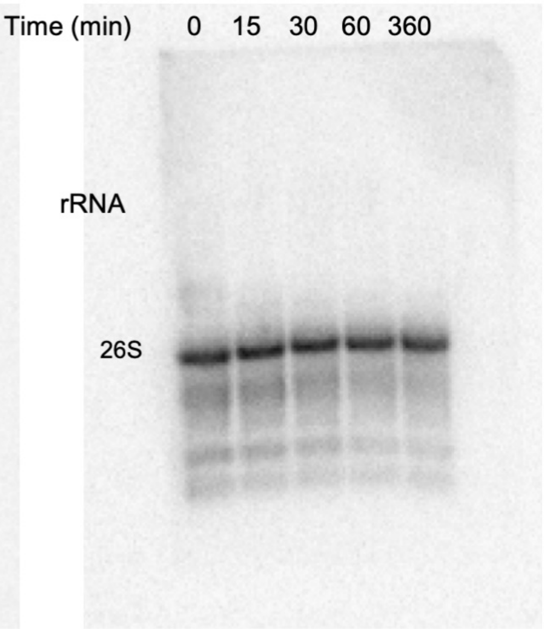

Uncropped 5D ITS1

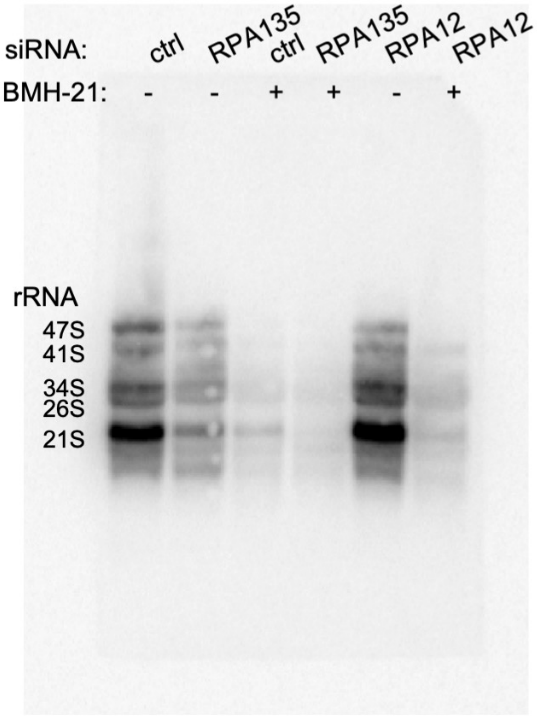

Uncropped 5D 28S

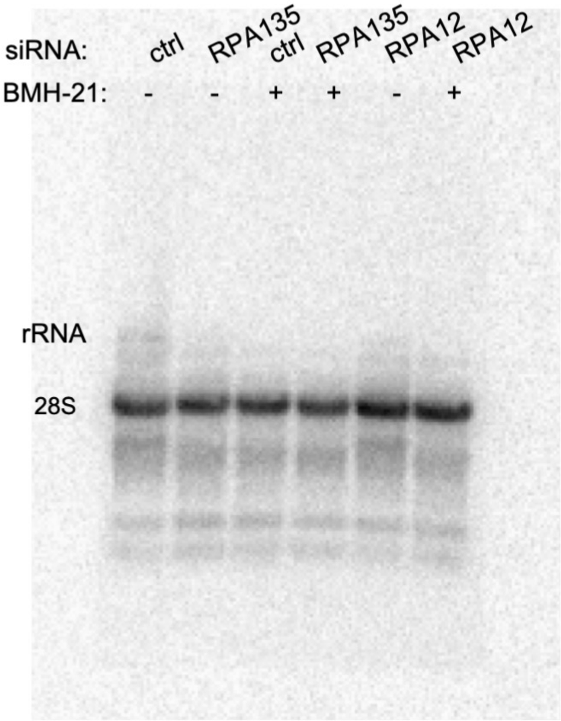

Supplement: S1 Raw images — (PDF) [file pone.0285660.s005.pdf]
